# The potential of durum wheat–chickpea intercropping to improve the soil available phosphorus status and biomass production in a subtropical climate

**DOI:** 10.1371/journal.pone.0300573

**Published:** 2024-05-13

**Authors:** Amira Souid, Wissem Hamdi, Boulbaba L’taief, Amal Attallah, Nourredine Hamdi, Mohammed O. Alshaharni, Mohamed Faouazi Zagrarni

**Affiliations:** 1 Higher Institute of the Sciences and Techniques of Waters, Gabes University, Gabes, Tunisia; 2 Biology Department, College of Science, King Khalid University, Abha, Saudi Arabia; 3 Laboratory of Composite Materials and Clay Minerals, National Center of Research in Materials Sciences, Soliman, Tunisia; University of Delhi, INDIA

## Abstract

The intercropping system is a promising approach to augmenting the soil nutrient status and promoting sustainable crop production. However, it is not known whether intercropping improves the soil phosphorus (P) status in alluvial soils with low P under subtropical climates. Over two growing seasons––2019–2020 and 2020–2021––two experimental fields were employed to explore the effect of durum wheat (Dw) and chickpea (Cp) cropping systems on the soil available P. A randomized complete block design was used in this experiment, with three blocks each divided into three plots. Each plot was used for one of the following three treatments with three replications: Dw monocrop (Dw-MC), Cp monocrop (Cp-MC), and Dw + Cp intercrop (CpDw-InC), with bulk soil (BS) used as a control. A reduction in the rhizosphere soil pH (-0.44 and -0.11 unit) was observed in the (Cp-MC) and (CpDw-InC) treatments over BS, occurring concomitantly with a significant increase in available P in the rhizosphere soil of around 28.45% for CpDw-InC and 24.9% for Cp-MC over BS. Conversely, the rhizosphere soil pH was significantly higher (+0.12 units) in the Dw-MC treatments. In addition, intercropping enhanced the soil microbial biomass P, with strong positive correlations observed between the biomass P and available P in the Cp-MC treatment, whereas this correlation was negative in the CpDw-InC and Dw-MC treatments. These findings suggested that Cp intercropped with Dw could be a viable approach in enhancing the available P through improved pH variation and biomass P when cultivated on alluvial soil under a subtropical climate.

## 1. Introduction

Phosphorus (P)––one of the three key elements controlling crop yields––is a crucial nutrient in the management of agro-ecosystems, is involved in an array of processes, such as photosynthesis and respiration, and is integral to several crop components, such as phospholipids [1]. Adequate P levels encourage vigorous biomass growth, promote early maturity, and increase water-use efficiency and grain yield. Consequently, P deficiency decreases vegetative growth and grain yields [2]. In Earth’s crust, P is the 11th most abundant element, and most soils may contain P pools that could be several thousand times higher than is necessary for crop growth [3]. Soil P is relatively stable in soil and moves very little compared to nitrogen (N) [4]. Although the total P content of soils may be large, only a small proportion might be available for plant uptake [5]. The lack of mobility and low solubility of P is governed by various reactions, including adsorption–desorption and precipitation–dissolution [6], and is strongly dependent on the pH value of the soil. In acid soils, P is mainly sorbed onto iron (Fe) and aluminum (Al) oxides and hydroxides, while in calcareous soils, it sorbs onto calcium (Ca) carbonates [6]. The highest availability of soil P is found in soils with neutral pH values of between 6 and 7.5. However, P can be immobilized in soil depending on the soil type, texture, amount of organic matter, and, most importantly, the presence of moisture. Semi-arid and arid soils contain much less available P than humid regions due to their low total P and high fixation of P [2]. However, due to limited global reserves of the phosphatic rocks used to produce P fertilizers, and because of their high costs, they need to be used wisely [7]. While applying mineral P fertilizer can enhance P availability in the soil, this generally may have costly and negative impacts on the environment due to the role of P in eutrophication [8]. For this reason, several studies have investigated methods of P fertilization that are more environmentally friendly and economically and biologically feasible in order to improve its resource efficiency and promote greater crop production. Previous studies have revealed that biological diversity in crop-growing environments can be improved by a recurrent succession of intercropping systems [9, 10]. Intercropping is the simultaneous cultivation of two or more crop species in the same field for a significant part of their growing periods [11]. This agricultural practice has the potential to enable sustainability in the management of the soil through crop diversification, improve the soil biological activity, and optimize nutrient cycling. Numerous types of intercropping systems have been practiced widely in many countries throughout the world, including wheat–maize [12], maize–fava bean [13], wheat–pea and maize–soybean [14], and legume–cereal [15]. In particular, cereal–legume is the most common type of intercrop cultivated worldwide and particularly in North African countries [16, 17]. Several studies investigating the effect of cereal–legume intercrops on rhizosphere soil P behavior have indicated that the legume species can solubilize the organic soil P through root-induced processes, such as acidification of the rhizosphere soil following the exudation of organic acids, including malate and citrate, and/or indirectly through microbial activity [18], thereby enhancing the P availability for the intercropped species, such as durum wheat (Dw) [19]. The benefits of cereal–legume intercrops for P uptake depend on the species arrangement [20] and soil P availability [15]. For this reason, several examples have been demonstrated in cereal–legume intercropping [15–18]. However, the results showing the facilitation of P uptake are not clear cut because, at the same time, the crop species are competing for other vital resources, such as water, nutrients, and light. Also, the mobilization of P by legume roots is not likely to be apparent if the soil has low P availability, especially in alkaline soils in the semi-arid region in the middle of Tunisia. The aim of this study was to investigate the effect of cereal–legume intercropping to increase P availability, P uptake, soil microbial biomass P, and yield in comparison to sole crops under the alkaline soils of the semi-arid region of Tunisia.

## 2. Material and methods

### 2.1 Study area

The study was carried out in the 2019–2020 and 2020–2021 growing seasons under field conditions. The experimental site was located in the Kairouan region of Middle-Eastern Tunisia (35°39’19”N, 10°01’57”E). The site was at an altitude of 68 m above sea level. The climate of Kairouan is subtropical, with mild winters and very hot, sunny summers. The annual mean temperature is 21.05°C, the average temperature of the coldest month (January) is 12.6°C, and that of the warmest month (August) is 30.7°C. The annual precipitation is 325 mm. Most of the precipitation occurs between October and March and has a unimodal distribution pattern.

### 2.2 Culture methods and plant growth

Samples were collected from the top 30 cm of the soil layer, and the soil physicochemical properties were determined prior to the beginning of the experiment. The samples were air-dried in the laboratory and sieved (2-mm mesh) prior to analysis. The field experiment was carried out using two crops commonly grown by Tunisia farmers––a chickpea (Cp) cultivar (*Cicer arietinum* L. cv. *Bochra*) and a Dw cultivar (*Triticum turgidum durum L*. *cv*. *Karim*). The randomized complete block design was used, involving three blocks, each divided into three plots. Each plot was used for one of the following three cropping systems with three replications: Dw monocrop (Dw-MC), Cp monocrop (Cp-MC), Cp + Dw intercrop (CpDw-InC). Bulk soil (BS) was used as a control. The area of each plot was 8 m^2^ (4 m× 2 m). The seeding density was 50 ± 5 seeds per m^2^ for the Cp-MC, 250 ± 20 seeds per m^2^ for the Dw-MC, and 30 ± 3 seeds per m^2^ for the Cp-InC + 150 ± 10 seeds per m^2^ for the Dw-InC. In both the sole and intercrop systems, the distance between the rows and plants for the Cp crop was 25 and 20 cm, respectively. The two crops were sown in the same row to maximize root proximity and Cp–Dw rhizosphere interactions. The seeding of both crops took place on September 18, 2019, and January 8, 2021. In terms of crop management, the intercropping was carried out without the application of fertilizers or herbicides and the plots were manually weeded. The plants were harvested at maturity––the Dw on June 15, 2020, and June 12, 2021, and the Cp on July 10, 2020, and July 12, 2021.

### 2.3 Soil and plant analysis

Ninety days after sowing, 12 plants were randomly harvested from the middle of each plot at the full-flowering growth stage and were grouped and treated as composite samples, one per plot. In the same way, at crop maturity, another 12 plants per plot were randomly harvested, excluding the border rows, in order to determine the yield from each treatment. The Dw shoots were cut at the shoot–root junctions, the Cp roots at the cotyledonary node. The roots and shoots of both species were oven-dried for 48 h at 65°C, then weighed. The P content (shoots and roots) was determined following the malachite green method following digestion in nitric (HNO_3_) and perchloric acids (HClO_4_). The plant biomass of each species was determined. In order to determine the land equivalent ratio (LER), Eq (1) was used.

LER=(ID/SD)+(Ic/Sc) Eq (1)

Where ID = intercropped Dw aboveground biomass; Ic = intercropped Cp aboveground biomass; SD = sole-cropped Dw aboveground biomass; and Sc = sole-cropped Cp aboveground biomass. The soil adhering to the Dw and Cp roots was sampled by brushing off the < 1–4-mm aggregates. The samples were then thoroughly mixed and pooled to make a composite sample for each plot. These total composite soil samples were air-dried, sieved (2-mm mesh), and analyzed using standard methods, as described in the following. The soil pH in water (soil: water ratio = 1:5) was determined from a soil suspension in deionized water using a pH meter. The electrical conductivity (EC, mS cm^–1^) of a 1:1 soil: water suspension was measured using a conductivity meter. The organic matter (OM) content was determined using the Walkley and Black procedure. The soil’s Ca carbonate (CaCO_3_) content was determined using the Horton and Newson method. The total N concentration was determined using the Kjeldahl method. The total P was determined by digestion using HClO_4_ and HNO_3_, and the available P was determined using the Olsen method. The soil exchangeable cations––Ca^2+^, magnesium (Mg^2+^), potassium (K^+^), and sodium (Na^+^)––were measured using an atomic absorption spectrophotometer (PerkinElmer, Inc., Shelton, CT, USA). The soil microbial biomass P was determined as the difference between the amount of inorganic P extracted by 0.5 (Spm) sodium bicarbonate (NaHCO_3_) (pH 8.5) from fresh soil fumigated with chloroform (CHCl_3_) and the amount extracted from unfumigated soil.

### 2.4 Statistical analysis

The plant and soil data corresponding to all the treatments were subjected to an analysis of variance using XLSTAT (Premium Version, 2017, Addinsoft, Long Island, NY, USA). The results obtained from the two cultivation seasons are reported as main effects and interactions. The means of the soil and plant parameters were compared using the Fisher test. The significance was determined at P < 0.05, and significantly different means are indicated by the use of different letters.

## 3. Results and discussion

### 3.1 Soil properties

The physicochemical properties of the topsoil (0–30 cm) prior to the beginning of the experiment are presented in Table 1. It was found that the proportion of loam (46%) was higher than the proportions of both clay (28%) and sand (26%). The topsoil was alkaline (pH = 8.5), the EC was 1.09 mS cm^–1^, and the OM content was low (2.8%). In terms of the agricultural conditions, there was a N deficiency (total N = 1. 87% and inorganic N as ammonium and nitrate [N-NH_4_^+^ + NO_3_^−^] = 5.8 mg N kg^−1^) and a P sufficiency (total P = 10.4 mg kg^−1^ and Olsen P = 8.86 mg P kg^−1^). The existence of N in small quantities was due to the low OM content and its removal through soil cultivation [4]. According to Zhang et al. [2] the soil would be classed as low in P (i.e., <600 mg kg^–1^). Generally, low P values can be related to the nature of the parent material and low phosphate (PO_4_)-fertilizer application [7]. Among the exchangeable bases, the soil was dominated by Ca^2+^ (226 mg kg^–1^), followed by Mg^2+^ (224.8 mg kg^–1^), whereas the K^+^ and Na^+^ cations were less abundant (190 and 125 mg kg^–1^, respectively). These results are consistent with those obtained by Amiri et al. [21].

**Table 1:** Main soil proprieties.

Proprieties	Values
Clay (%)	28
Loam (%)	46
Sand (%)	26
pH water	8.5
EC (mS.cm^-1^)	1.09
CaCO_3_ (%)	2.1
Organic matter OM (%)	2.8
Total-P (mg kg^−1^)	10.4
Olsen-P (mg kg^−1^)	8.86
Total- N (%)	1. 87
N-NH_4_^+^ + NO_3_^−^ (mg kg^−1^)	5.8
Basic cations (mg kg^−1^)	
Ca^2+^	226
Mg^2+^	224.8
K^+^	190
Na^+^	125
C/N	8.41

### 3.2 Available soil phosphorus

**Fig 1:**
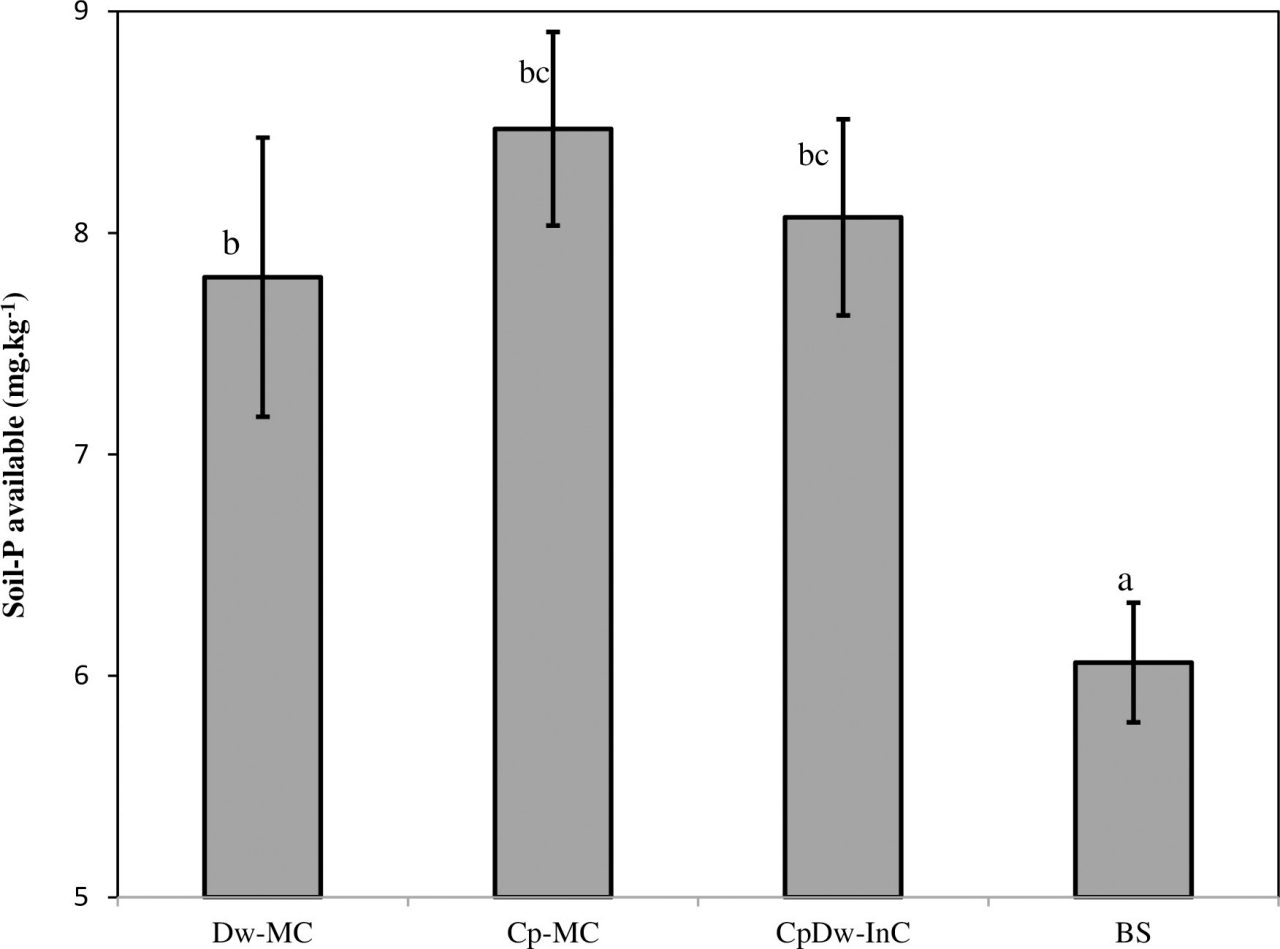
Mean fraction of soil-P available values across all treatments during the two cropping seasons. BS: Bulk soil, Dw-MC: mono-cropped durum wheat, Cp-MC: mono-cropped chickpea, CpDw-InC: durum wheat intercropped with chickpea. Values are the mean of three replicates for the two cropping seasons. The error bars represent the standard error of the mean. Different letters indicate a s significant difference at p < 0.05.

The availability of rhizosphere soil P (i.e., the Olsen P) over the two cropping seasons (Fig 1) increased significantly, by 22.3% for the Dw-MC, 28.4% for the Cp-MC, and 24.9% for the CpDw-InC compared to the BS. The available P was higher with the Dw and Cp grown as intercrops and Cp as a sole crop than Dw as a sole crop and the BS. Similar results had previously been reported for CpDw-InC field experiments conducted in fertile soils [22]. Recent studies have shown the benefits that come from intercropping Dw with Cp due to the latter’s facilitative mechanisms (i.e. exudation of phosphatases and carboxylates, release of protons and organic acids, and/or indirectly through microbial activity), which are responsible for increasing the available P through rhizosphere acidification during P-deficient rhizosphere N fixation in alkaline soil [15, 17]. Similarly, Chen et al. [18] reported that Cp roots can exude several acid components that can acidify the Cp rhizosphere and increase P availability through the dissolution of P minerals [23]. This finding supports the idea that the formation of Cp cluster roots plays a significant role in acquiring P from soils. Also, Tang et al. [15] assumed that, where soil conditions are limited, as in P-deficient alkaline or calcareous soils, these positive interactions are invaluable [15].

### 3.3 Effect of intercropping on pH

**Fig 2:**
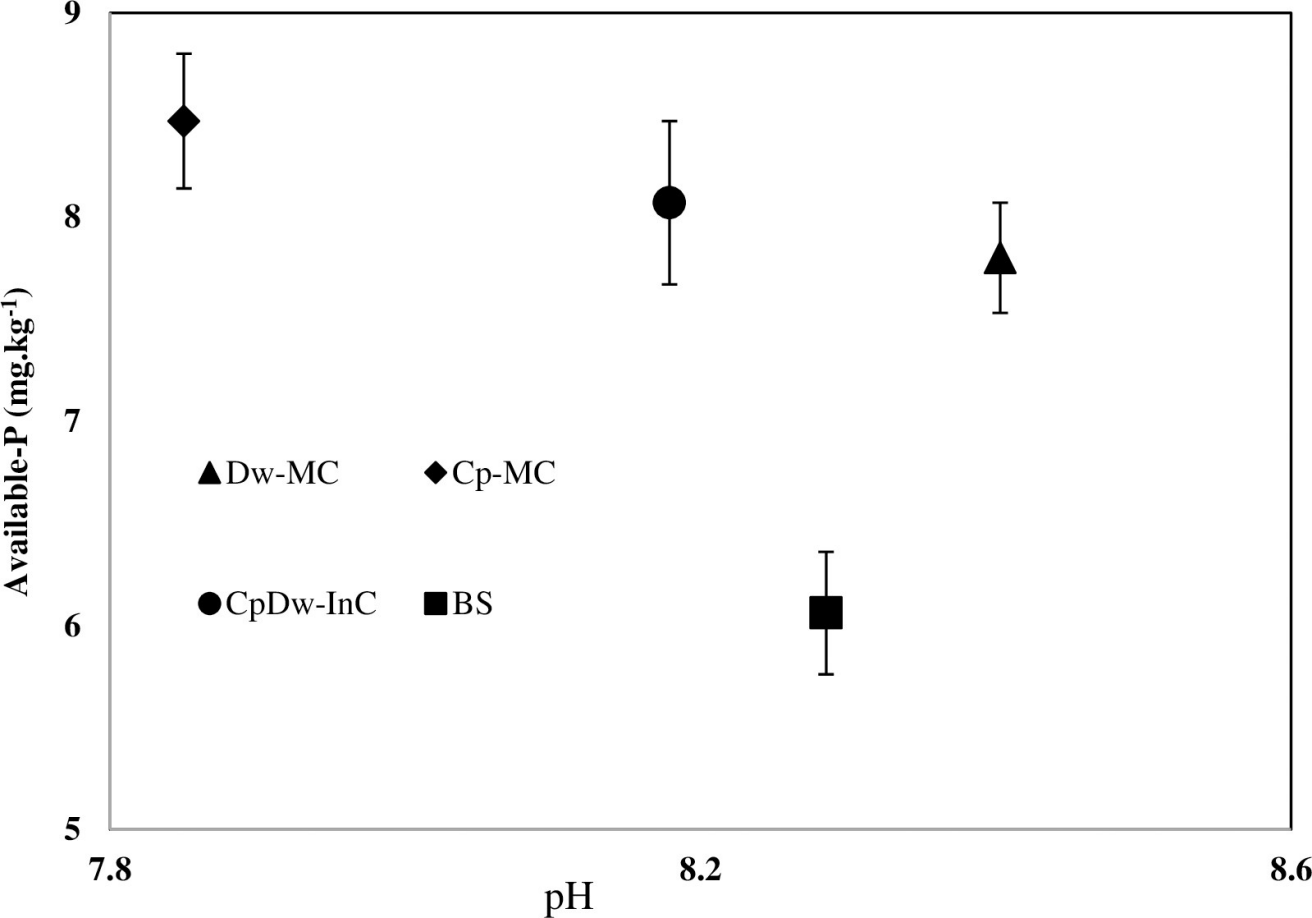
Effect of pH variation (mean values) on P-available (mean values) across different treatments during the two cropping seasons. BS: Bulk soil, Dw-MC: mono-cropped durum wheat, Cp-MC: mono-cropped chickpea, CpDw-InC: durum wheat intercropped with chickpea. Values are the mean of three replicates for the two cropping seasons. The error bars represent the standard error of the mean.

The variation in pH under the different treatments over the two cropping seasons is shown in Fig 2. It was found that Cp-MC and CpDw-InC acidified the rhizosphere, resulting in a decrease in pH of -0.44 and -0.11, respectively, compared to the BS. It has been determined that Cp roots can exude several acid components or hydrogen ions (H^+^), which causes acidification of the Cp rhizosphere. By contrast, with a Dw-MC, the pH can increase by 0.12 compared to that of the BS. These results can be explained by the preferential uptake of NO_3_^−^ as the N source by wheat plants. Similar results have been reported by previously [15, 24]. Latati et al. [16] discovered an exudation of malate and citrate by wheat plants, but to a lesser extent than legumes. More-recent studies have also found an exudation of malate and citrate by wheat plants to a lesser extent than legumes, but which did not significantly reduce pH values [15, 23]. For the CpDw-InC, the reduction in soil pH enhanced cation removal from the soil due to over-yields from the intercropping system. Also, Uher et al. [24] stated that root-induced pH changes are mainly influenced by H^+^/hydroxide (OH^–^) exudation, depending on the cation–anion balance, which is driven mainly by N nutrition and changed chemical properties in the soil, and thus the surface charge of the minerals influencing the partitioning of P ions. It can also be seen that the decrease in pH values increased the available P in the rhizosphere of the CpDw-InC (by 28.45%) and Cp-MC (by 24.9%) (Fig 2). This supports the previous understanding that acidification can increase P availability in neutral to alkaline soils [15, 25]. Considering the rhizosphere of the Cp-MC, the relationship between the available P and pH indicated that root-induced pH changes resulted from a prominent rhizosphere process that drove P availability. In the CpDw-InC, there was no significant correlation between pH and available P (P < 0.05). These findings may suggest that the effect of pH on the available P has been masked by the effect of the change in pH, resulting in a decrease in P availability over a significant distance from the plant roots. The acidification of the rhizosphere by exudates from the Cp likely benefited the intercropped Dw by increasing P availability through the dissolution of P minerals [26]. In addition, Schwerdtner et al. [20] reported that Cp species secrete higher levels of phosphatase in P-deficient soil conditions through their root systems. These results support the idea that cluster-root formation plays a significant role in acquiring P from alluvial soils under semi-arid climates. However, previous studies have revealed that a decrease in alkaline soil pH values can alter P availability through either the dissolution of P minerals, such as Ca_3_ (PO_4_)_2_, or the desorption of PO_4_^3–^ ions bound to soil constituents [1]. Moreover, legume species, such as Cp, take up more Ca^2+^ than monocotyledonous (grass) species, such as Dw [27]. This may explain the differences between Dw and Cp when considering the root-induced changes in pH and P availability in their respective rhizospheres.

### 3.4 Phosphorus in the soil microbial biomass

**Fig 3:**
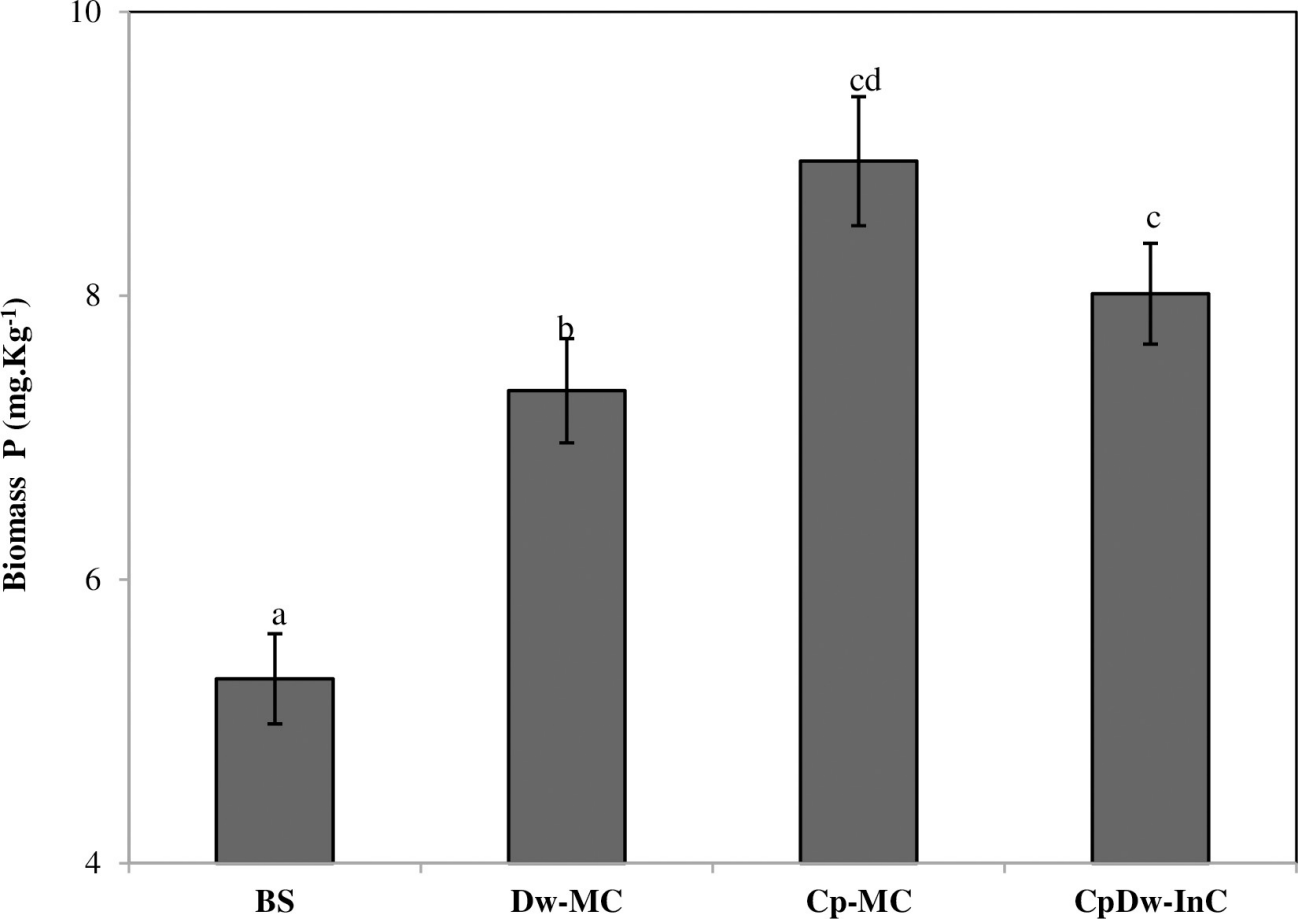
Mean biomass-P values across all treatments during the two cropping seasons. Values are the mean of three replicates for the two cropping seasons. BS: Bulk soil, Dw-MC: mono-cropped durum wheat, Cp-MC: mono-cropped chickpea, CpDw-InC: durum wheat intercropped with chickpea. The error bars represents the standard error of the mean. Different letters indicate a significant difference at p < 0.05.

**Fig 4:**
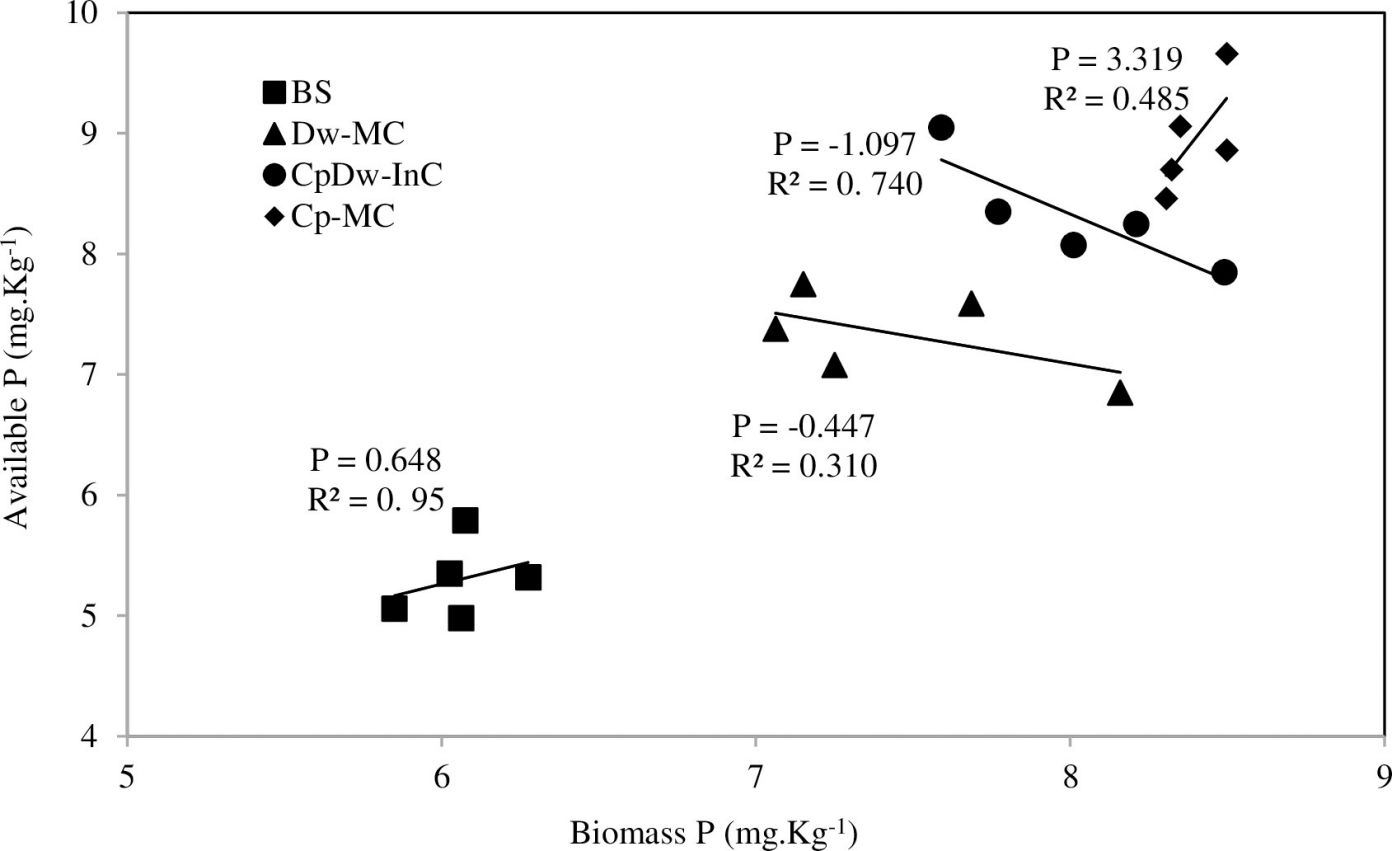
Relationship between average available-P and the soil biomass-P in the rhizosphere across all treatments during the two cropping seasons. BS: Bulk soil, Dw-MC: mono-cropped durum wheat, Cp-MC: mono-cropped chickpea, CpDw-InC: durum wheat intercropped with chickpea. Lines represent the relationship between available-P and the soil biomass-P in BS (slope = 0.648, p <0.05, R^2^ = 0.95), Dw-MC (slope = -0.447, p <0.05, R^2^ = 0.310), Cp-MC (slope = 3.319, p<0.05, R^2^ = 0.485) and in the CpDw-InC (slope = -1.097, p < 0.05, R^2^ = 0.740).

The P in the soil microbial biomass (i.e., the biomass P) is an important part of the pool of soil P available for crop growth. Our results indicate that the microbial biomass P increased significantly (P > 0.05), by 40.7%, 33.8%, and 27.6%, in the Cp-MC, CpDw-InC, and Dw-MC relative to the BS over the two cropping seasons (Fig 3). Our findings support previous studies that indicated that Cp plant species can influence the biomass P through the abundance, activity, and composition of the soil decomposer communities in their rhizospheres [19]. Our results also demonstrated that the Cp-MC and CpDw-InC improved the biomass P compared to the Dw-MC, indicating that the Cp may have utilized more soil organic P and recovered cycled nutrients, and that soil fertility can positively affect the microbial biomass [19]. In addition, there was a positive correlation between the biomass P and available P over the two cropping seasons (Fig 4) in the Cp-MC treatment (r^2^ = 0.485, slope = 3.31, P > 0.05) and a negative correlation in the CpDw-InC (r^2^ = 0.74, slope = -1.097, P > 0.05) and Dw-MC (r^2^ = 0. 31, slope = -0.447, P > 0.05) treatments. However, there was no significant correlation in the BS. The contrasting results found between the treatments may indicate that different processes, such as predation or phage dynamics, were also involved in the rhizosphere [28]. In addition, the increase in biomass P in the CpDw-InC treatment appeared to be the result of microbial growth in the soil rhizosphere. This may be due to differences in resource allocation in the microbial communities, with microorganisms around the Cp roots improving growth and those around the Dw roots storing P, even when the Cp and Dw were intercropped [29]. Contrastingly, Chenene et al. [30] showed that the organic soil P pool might account for up to 65% of the total P in the organic soil reservoir and that the microorganisms can mineralize this significant fraction and enhance the available P in soil solution.

### 3.5 Growth performance of cereal–chickpea intercropping

**Fig 5:**
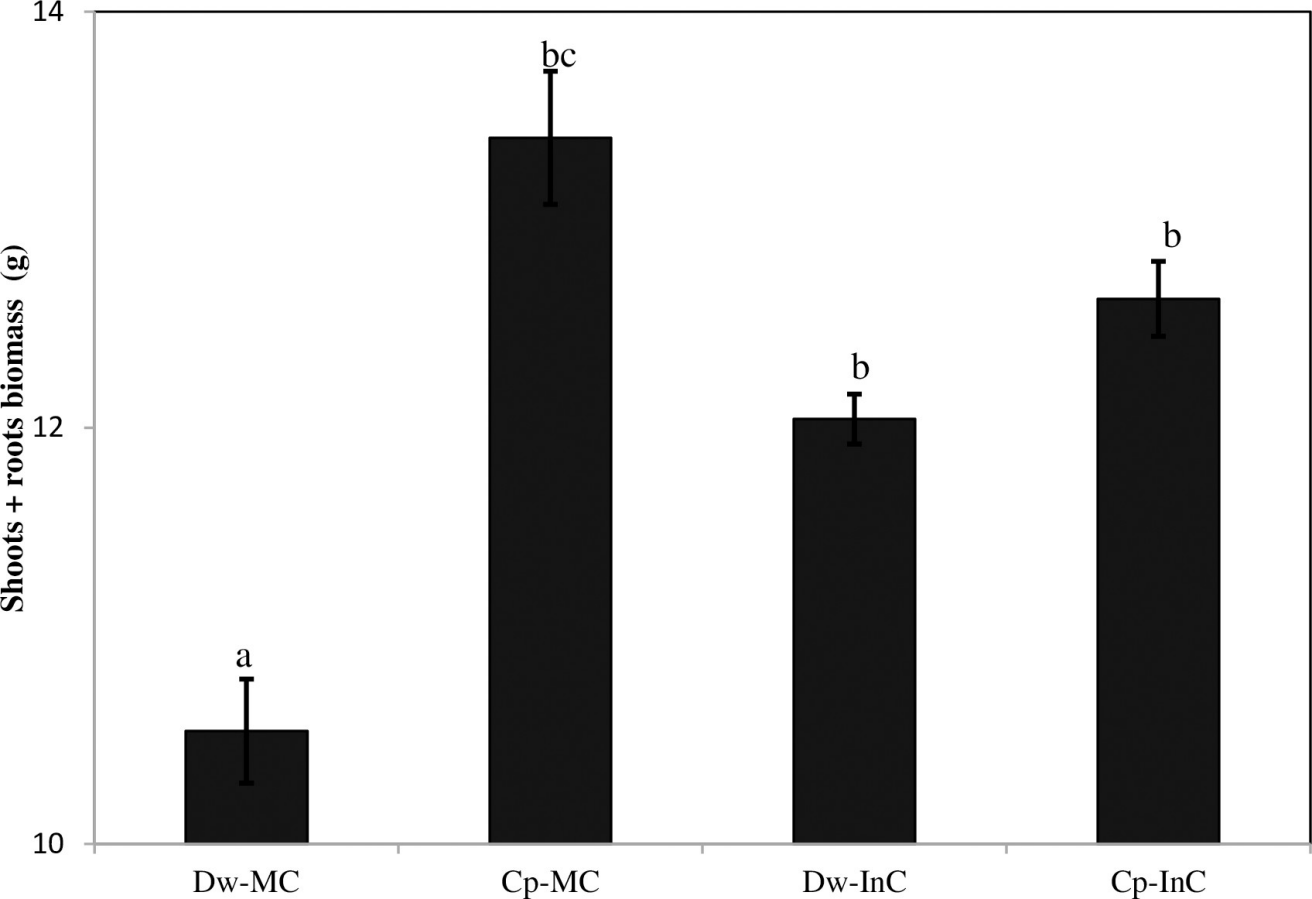
Mean plant growth (shoots + roots) biomass values across all treatments during the two cropping seasons. Dw-MC: mono-cropped durum wheat, Cp-MC: mono-cropped chickpea, Dw-InC: durum wheat intercropped with chickpea, Cp-InC: chickpea intercropped with durum wheat. Values are the mean of three replicates for the two cropping seasons. The error bars represent the standard error of the mean. Different letters indicate a significant difference at p < 0.05.

**Fig 6:**
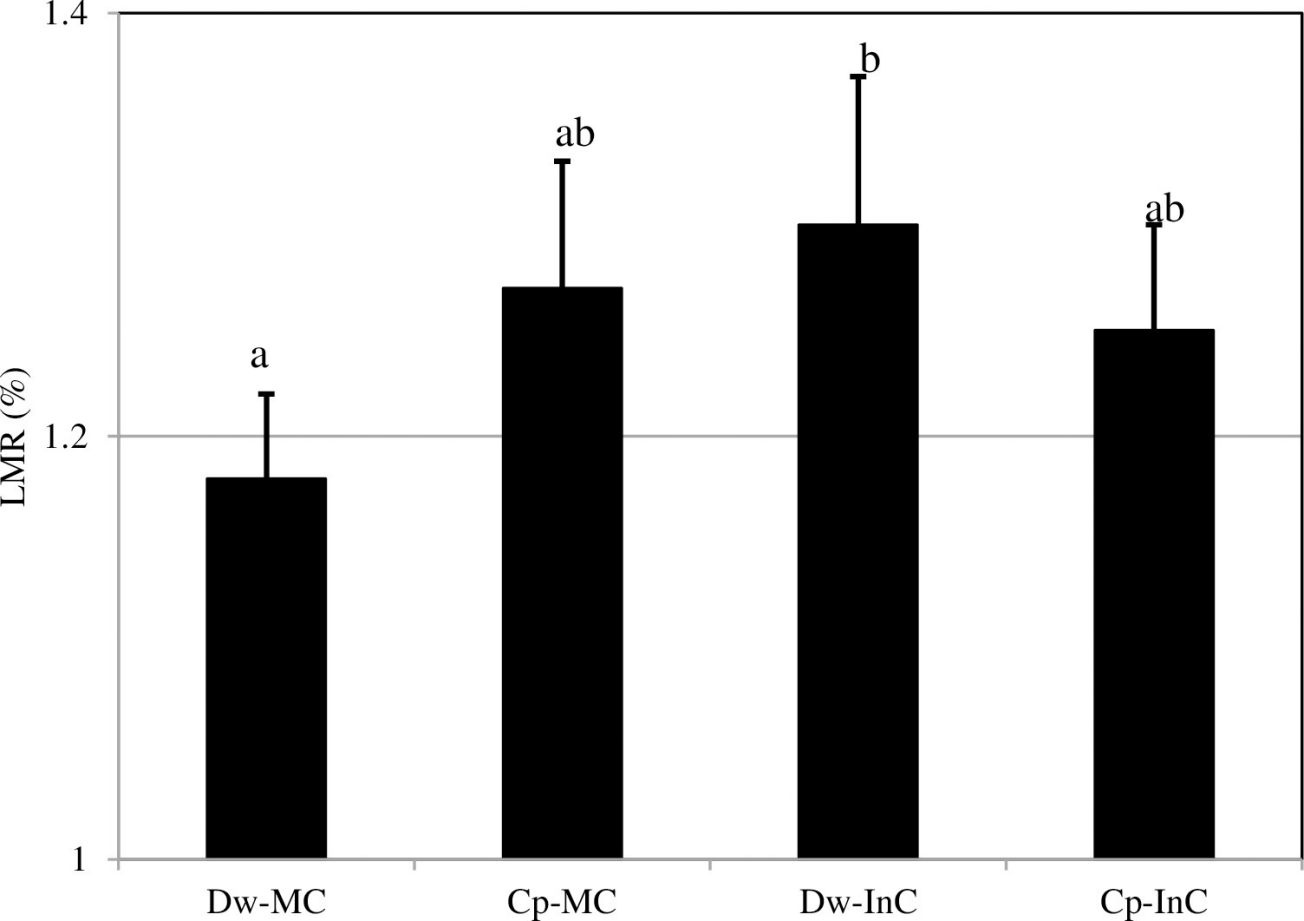
Average LER values under different cropping systems over the two cropping seasons. Dw-MC: mono-cropped durum wheat, Cp-MC: mono-cropped chickpea, Dw-InC: durum wheat intercropped Cp-InC: chickpea intercropped. The horizontal bars represent 95% confidence intervals of estimates.

Overall, our results from the two cropping seasons (Fig 5) showed a significant increase (14.23%) in the dry (shoots + roots) weight for the Dw intercropped with Cp compared to the Dw-MC in a P-deficient and alkaline soil. However, when the Cp was intercropped with the Dw, the dry weight of its shoots and roots was significantly decreased (5.7%). Our data are similar to those from previous field studies in the Mediterranean region that have investigated the impact of intercropping various legume–cereal systems on plant growth [31]. These authors also confirmed that the shoot and root biomasses were significantly lower when Cp was intercropped with Dw. Indeed, the change in biomass weight could be attributed to the effect of the N fixed by the Cp crop in the intercrop system [30]. However, the high Cp biomass weight observed in the Cp-MC treatment compared to the intercropping system could be attributed to competition between the crops for growth resources, such as light, nutrients, and water [32]. This suggests that competition between the Dw and Cp in the intercropping treatment significantly affected Cp growth compared to in the Cp-MC. Previous studies have reported that differences in the depth of the roots, lateral root spread, and root density may result from competition for nutrients between the component crops in an intercropping system [30]. Also, a fertile soil rhizosphere has a higher supply of resources and leads to lower competition between plants than less fertile ones, where Cp can enrich the soil nutrient base through the fixation of atmospheric N into the soil [33, 34]. According to Shevchenko et al. [35], shading the Cp species component in an intercrop system can affect N_2_ symbiotic fixation and photosynthesis, especially during the full flowering stage. This may offer opportunities for sustaining the enhancement of plant biomass in intercropped species [36]. This kind of system improves the health of the plants, reduces the spread of disease, and enhances plant growth [37]. Furthermore, intercrop systems can suppress weeds, thus reducing the competition for water and nutrients between the cultivated plants and the weeds, and favoring the growth of the cultivated plants. Several researchers have observed a decline in Dw growth due to competition with legumes at the end of the plant growth period (e.g. [30, 38]). During this period, favorable growth conditions are created for the plants in the lower crop level. In addition, our study showed that the LER values were significantly greater than 1 (Fig 6), ranging from 1.18 to 1.3, in almost all cases under the different treatments over the two cropping seasons, and were more significant for the intercropping, which is interpreted as the advantage of the intercropping system over the monocrop system. Our results strongly coincided with the definition of the LER where the combination of component species in the intercropping system is more productive than those same species grown as monocrops. Additionally, our results are in agreement with those of Rodriguez et al. [22], who reported an increase in wheat yield when intercropped with Cp over three cropping seasons, as well as other studies that have reported the benefits of cereal–legume associations on grain yield [16, 17], who reported an increase in the yield of wheat associated with Cp compared to its sole crop. However, some previous studies have reported that LER values of less than or equal to 1.0 indicate no difference in yield between intercropped and mono-cropped species, while any value greater than 1.0 indicates a yield advantage in intercropping [39, 40] confirmed that the LER can be used as a measure of relative yield advantage. For example, a LER value of 1.2 indicates that an area planted with a monoculture would need to be 20% larger than an area planted with an intercrop to produce the same combined yield.

### 3.6 Root and shoot biomass phosphorus concentrations

**Fig 7:**
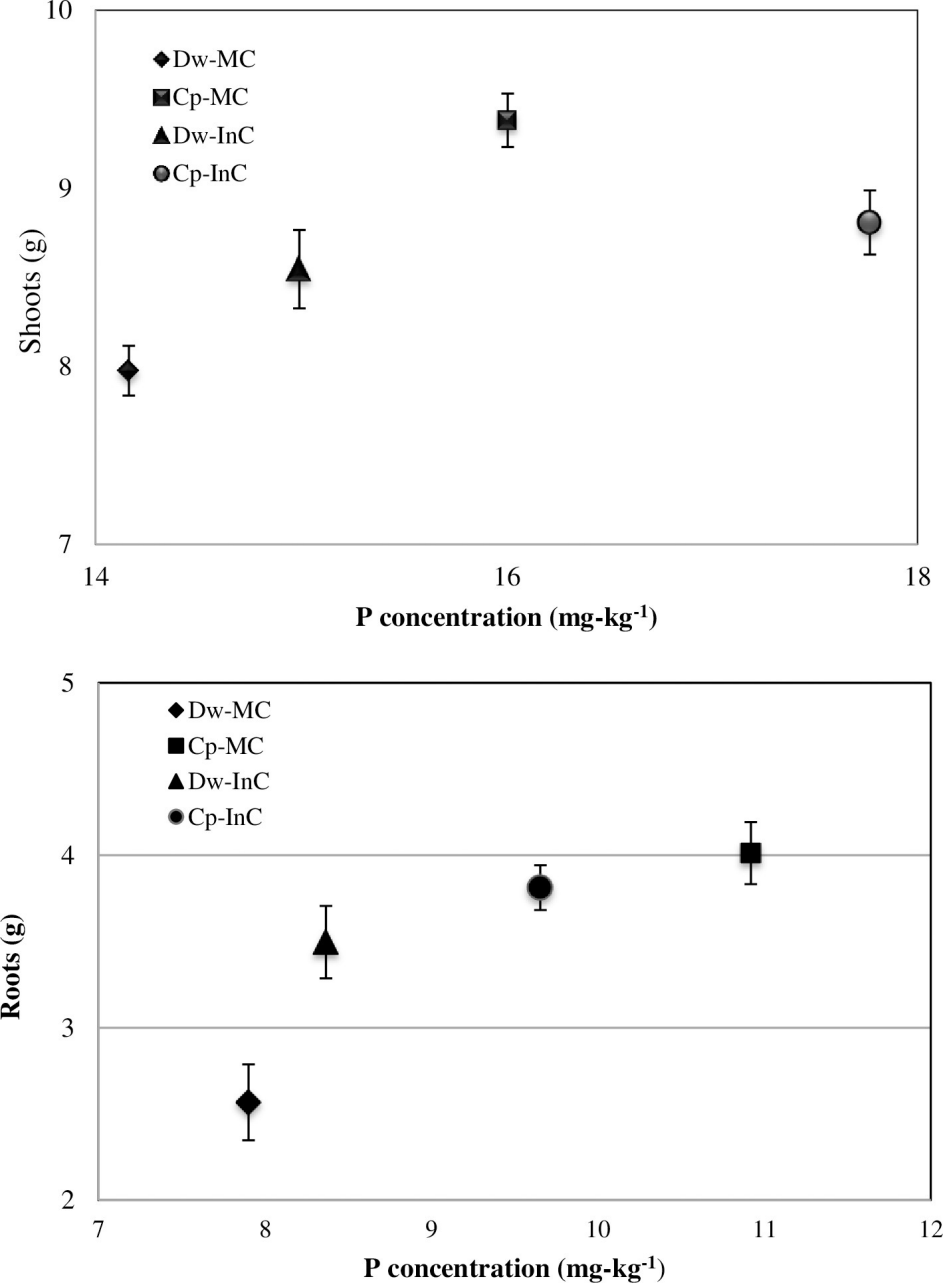
Mean values of roots and shoots biomass and P contents of all cropping systems during two seasons. Dw-MC: mono-cropped durum wheat, Cp-MC: mono-cropped chickpea, Dw-InC: durum wheat intercropped with chickpea, Cp-InC: chickpea intercropped with durum wheat.

Fig 7 shows that the P concentrations in the dry-weight root and shoot biomasses at the complete vegetation growth stage varied significantly under the intercropping system compared with the monoculture systems over the two cropping seasons (P < 0.05), with the P concentrations in the shoots and roots following the order Cp-MC (9.38–4.01) > Cp-InC (8.8–3.81) > Dw-InC (8.54–3.49) > Dw-MC (7.97–2.56), indicating that P is an essential resource for Cp, and that the P fixed by this plant can be used by the Dw in the intercropping system during its growth stages. This may be due to the more-complex biological diversity associated with intercropping systems, with P being transferred to the soil via ions and root exudates, further facilitating the accumulation and decomposition of the soil P pool [5]. However, Chen et al. [18] reported that Cp–mixed intercropping increases P availability to provide higher P levels for utilization by the adjacent crop, thus providing a growth advantage for the intercropped plant. Our results revealed that, when we compared the Dw-MC to the Dw-InC, the P concentrations in the shoots and roots increased by 6.7% and 5.41%, respectively, whereas they declined by 9.2% and 13.4%, respectively, in the Cp-MC compared to the Cp-InC. This indicates that higher soil fertility might be expected to result in greater plant growth, resulting in the exudation of diverse ranges of organic acids by Cp, thereby facilitating P assimilation [41]. Thus, our findings suggest that intercropping can result in a large amount of P being added to the rhizosphere soil and an increase in nutrient accumulation [42]. Also, our results suggest that P was the limiting element in determining Dw and Cp productivity in the intercropping and monoculture systems in alkaline soils in the arid region of southern Tunisia.

## 4. Conclusions

Consistent with the aim of this study, we observed that Cp intercropped with Dw resulted in significantly enhanced available P and biomass P performance in the subtropical climate over the two growing seasons. In general, Dw intercropped with Cp displayed superior agronomic performance compared to Dw alone. Collectively, a reduction in RS‒pH and an increase in microbial activity appeared to be the most important determinants of the status of the soil available P and improved biomass P production when Dw and Cp were cultivated as intercrops in the alluvial soil under semi-arid climatic conditions. Our results reaffirm the advantages of intercropping Cp and Dw in terms of growth, yield, and roots and shoots at the full vegetation stage. The available P and biomass P in the rhizospheres of the Dw and Cp plants were significantly higher than in the BS, ranking in the order Cp-MC > CpDw-InC > Dw-MC. This work significantly improves in our understanding of agricultural production in a semi-arid climate, particularly in the context of climate change and expanding global populations in these geographic regions, where food security is anticipated to become a challenge in the future. However, given the complexity of soil P availability, which largely depends on the soil properties and agricultural practices, additional research is necessary to obtain an understanding of the interactive relationships between soil P, agricultural practices, and their history and management.

## Supporting information

S1 File Minimal study dataset used to create graphs and analyze the data reported and interpreted in the manuscript. (XLSX)
